# The quality changes and microflora analysis of commercial instant soya milk

**DOI:** 10.1002/fsn3.371

**Published:** 2016-05-19

**Authors:** Xiaohong Ma, Xinzhong Hu, Liu Liu, Xiaoping Li, Zhen Ma, Jiahui Chen, Xue Wei

**Affiliations:** ^1^ College of food engineering and nutritional scienceShaanxi Normal UniversityXi'anShaanxi710119China

**Keywords:** Microbial analysis, protein denaturation, sensory analysis, soya milk, viscosity

## Abstract

Soya milk can be easily contaminated because of its rich nutritional profile and simple package form, which thus may lead to short shelf life and has been considered as a public health concern. The objective of this study is to investigate the changes of sensory quality, viscosity, pH values, bacteria, and protein denaturation in soya milk samples which were stored for 0, 4, 8, 12, and 24 h at 25 ± 2°C. The results showed that the sensory properties were on the decline along with the storage time. The viscosity value increased from 1.61 Pa.s to 2.50 Pa.s, while the pH value decreased from 6.87 to 6.61. In addition, the number of total bacteria and *Lactobacillus* increased and the protein in soya milk denatured continually. The 16S rDNA sequence analysis indicated that the main microbial strains were *Rummeliibacillus*,* Acinetobacter, Enterobacter, Phyllanthus, Bergia*,* Zhihengliuella*, and *Nesterenkonia*. In this study, statistics in producing, packaging, and stocking process of soya milk were given, which provided experience to controlling soya milk.

## Introduction

Soya milk has been a traditional drink for breakfast in Southeast Asia. It is made from soaked soybeans by grinding, heating, and filtering. Soybean‐based products are very popular and have attracted worldwide interest. China, the largest importing country to domesticate soybeans and a major global soybean grower and consumer, has extensive distributions of soybean accessions (Li et al. 2014). Recently, researchers are interested in the physiologically beneficial phytochemicals from soybeans and soya products (Jooyandeh 2011). Isoflavones, an important component of soybeans and soya products, has been reported to protect against cardiovascular diseases, breast cancer, prostate cancer, testicular cancer, uterine cancer and other hormone‐dependent cancers, and osteoporosis (Patisaul and Jefferson 2010). In addition, soya bean and soya products are good sources of phytosterols, polyamines, and tocopherols, which has been reported to help control cholesterol, protect against various types of injuries, and has showed strong antioxidant activity (Marangoni and Poli 2010; Bae et al. 2008; Niki and Noguchi 2004). Soya milk is the biggest soya‐based product consumed in the world, not only because of its potential health benefits but also as an alternative to cow milk targeting for lactose‐intolerant individuals, those allergic to milk proteins, or those avoiding consuming milk for other reasons (Reilly et al. 2006). Soya milk occupies an important position in China health program for breakfast. Commercial soy beverages in glass bottles are sterilized batch wise in retorts or continuously in a hydrostatic sterilizer to achieve commercial sterility (Kwok and Niranjan 1995). At present, soya milk in the market basically has instant soy milk powder and instant soya milk. When soy milk powder is dissolved in hot or boiled water, the protein contained was denatured and the reconstituted soya milk has beany flavor.

In China's health project for breakfast, plastic packaging of soya milk has been widely used. Soya milk, which is rich in nutrition, may promote bacteria growth when contamination happens. *Lactobacillus* was reported to be one of the most common spoilage and pathogenic microorganisms presented in soya products (Odu and Egbo 2012). The contamination of these microorganisms in soya milk during processing or stocking will result in a potential risk for public health and a reduced shelf life. So, the contamination of soya milk should be tightly controlled and monitored.

In this study, we aimed to identify the changes of sensory perception, pH, viscosity from soya milk samples kept at 25 ± 2°C for 0, 4, 8, 12, and 24 h. Moreover, bacteriological parameters were also monitored during the experimental period. The results may help both the producers and consumers to better understand the quality control of soya milk.

## Materials and Methods

### Soya milk samples treatments

Soya milk samples were purchased from Xi'an Commercial soya milk. The soya milk samples without opening the package were stored for 0, 4, 8, 12, and 24 h at 25 ± 2°C, respectively.

### Methods

#### Sensory analysis of soya milk samples

To present the samples to the panelists, soy milk was placed in white cups, with blind codes with two digits. Each panelist in his/her white‐lighted booths received his/her samples in a random presentation order and answered the questions at their computers. Between the samples given, the mouth was cleaned with water. To analyze flavor and texture, the panelists tasted an adequate amount of food samples (Ghosh and Chattopadhyay 2012). All the food samples were presented by pair evaluated in color, bean flavor, beany, quality, flavor, and overall acceptability. The principle of Quantitative Descriptive Analysis (QDA) is based on the ability to train panelists to measure specific attributes of a product in a reproducible manner to yield a comprehensive quantitative product description amenable to statistical analysis. Panelists were recruited from the staff members of College of Food Engineering and Nutritional Science, Shaanxi Normal University with a focus to identify key product attributes. This group of panelists is then trained to reliably identify and score product attributes. As panelists generate the attribute terms, the resulting descriptions are meaningful to consumers, and thus, analysis provide information amenable to modeling predictions of consumer acceptability. QDA results are analyzed statistically and then represented graphically.

#### Measurement of pH

The pH value was determined in triplicates for each sample by a digital pH meter (PHSJ‐3F, ex Electric Chemical; Shanghai, China).

#### Viscosity analysis

The viscosity of soya milk sample was measured by viscometer (RVDV‐II+Pro; Brookfield, USA). All tests were performed at 5.0 ± 0.02°C. The samples were presheared for 5 sec at a controlled shear rate of 2001/sec to create a dispersion of uniform properties allowed to equilibrate for 30 sec before measuring intrinsic viscosity (Purwandari et al. 2007). The measurements were then performed at a constant shear rate of 25.81/sec for 60 sec according to previous report (Burkus 2003). The viscosity values were monitored.

#### Bacteria and *Lactobacillus* enumeration

Colony‐forming units (CFUs) of bacteria and molds were counted using a plate‐counting method. Soya milk sample (1 mL) was dispersed in 9 mL of sterile saline solution and oscillated for 1 min to achieve a 10‐fold dilution. A sterile cotton wool was wetted by sterile saline, the inside wall of a soya milk bottle cap was wiped, and then 10 mL sterile saline was added to the test samples. The dilution with sterile saline solution was repeated twice to achieve three continuous dilutions. A 0.1 mL aliquot from each serial dilution was spread over an agar plate with plate count agar (PCA) medium for molds, and over a plate of de Man, Rogosa, and Sharpe (MRS) for *Lactobacillus*. The plates were incubated at 37°C for 48 h. CFUs, which varied from 10 to 100 for molds and from 30 to 300 for bacteria, were counted.

#### Isolation of total microbial DNA

For microbial genomic DNA extraction, 10 mL of each sample were seeded in 100 mL of basic culture medium and incubated overnight in a shaker. Total DNA was isolated by using Bacteria Gen DNA Kit (Cwbio, Beijing, China) according to the manufacturer's instructions.

#### DGGE analysis

##### PCR amplification of 16S rDNA sequences

Genomic DNA was used as a template in PCR amplifications of the V3 region of the bacterial 16S rRNA gene, using the universal primers F341‐GC (5‐CCTACGGGAGGCAGCAG‐3′) and R534 (5‐ATTACCGCGGCTGCTGG‐3′) as reported by Zijnge et al. (2003), V3–V5 region of the bacterial 16S rDNA gene, using the universal primers F341‐GC(5‐CCTACGGGAGGCAGCAG‐3′) and R926 (5′‐CCGTCAATTCCTTTRAGTTT‐3′) as reported by Fujimoto et al. (2003). All GC primers contained a 40 bp GC‐clamp sequence at their 5′ end to prevent the complete denaturation of amplicons, GC‐clamp sequence is: 5‐CGCCCGCCGCGCCCCGCGCCCGTCCCGCCGCCCCCGCCCG‐3′ as reported by Yu and Orrison (2004). PCR was performed in 50 *μ*L reaction volumes using a Taq DNA polymerase master mix (cwbio, Beijing, China) with ~100 ng of each DNA sample as a template and 0.2 mmol/L of each primer.

##### Electrophoretic and identification of bands

Denaturing gradient gel electrophoresis (DGGE) was performed using a DCode apparatus (Bio‐Rad, Richmond, CA) at 60°C and employing 8% polyacrylamide gels with a denaturing range of 40–60% for total bacteria. Electrophoresis was performed at 75 V for 16 h and 130 V for 4.5 h for bacteria. Bands were visualized under UV light after staining with ethidium bromide (0.5 mg mL^−1^) and photographed.

Bands in the gels were identified by sequencing. Bands were excised from the gels and set to sequence (Sangon Biotech, Shanghai, China). The identity of the sequences was determined by the BLASTN algorithm in the GenBank database (http://www.ncbi.nlm.nih.gov/BLAST/).

### Pyrosequencing analysis

#### 16S rDNA gene amplification conditions

Amplifications were performed using the following PCR conditions: V3 region primers conditions: 94°C for 2 min, 30 cycles of 94°C for 30 sec, 60°C for 30 sec and 72°C for 30 sec, and a final extension step at 72°C or 2 min. V3–V5 region primers conditions: 94°C for 2 min, 30 cycles of 94°C for 30 sec, 50°C for 30 sec and 72°C for 30 sec, and a final extension step at 72°C for 2 min. The sequences obtained were uploaded at the NCBI Sequence Read Archive.

#### Sequence treatment and bioinformatics analysis

The MOTHUR program was also used to perform the Fast UniFrac test, which was employed to compare the phylogenetic structure of the libraries and to generate the Venn diagrams. A neighbor‐joining tree was constructed with representative sequences of each Optical Transform Unit (OTU) selected by MOTHUR. These sequences were compared against the RDP database using the Seqmatch option to select for the nearest neighbors. All sequences were then aligned using MEGA 5.0 software (Tokyo, Japan) (Tamura et al. 2011) and the Jukes–Cantor model.

#### Protein analysis with SDS–PAGE

Buffer A: 0.2 mol/L Na_2_HPO_4_ (31.2 g Na_2_HPO_4_. 12 H_2_O add distilled water to 1000 mL and mixed well); buffer B: 0.2 mol/L NaH_2_PO_4_ (35.6 g NaH_2_PO_4_.2 H_2_O add distilled water to 1000 mL and mixed well); Phosphate buffer (PB): 0.2 mol/L PB (pH 7.4, 19 mL buffer A added to 81 mL buffer B and mixed well).

For total protein extraction, each soya milk sample (10 g) was transferred into a hydrolysis tube with 100 mL of PB, and stir‐extracted for 60 min on ice, and then centrifuged (15,000 × g/min, 45 min) at 4°C. The supernatant was centrifuged again, and the supernatant was saved as soya milk protein samples.

#### SDS–PAGE analysis

Protein samples were added with 2% of 2‐mercaptoethanol and heated in boiling water bath (100°C) for 3 min. The samples (8 or 16 *μ*L) were loaded to SDS‐PAGE with 16% separating gel according to the method by Schägger (2006). The samples were electrophoresed at constant voltage of 30 mV until all samples entered into the stacking gel, and then at constant voltage of 100 mV until end. After electrophoresis, the gel was fixed with a solution of 100 mmol/L ammonium acetate dissolved in methyl alcohol/acetic acid (5/1, v/v) for 2 h. After fixing, the gel was stained with 0.025% (w/v) Coomassie Blue G‐250 in 10% (v/v) acetic acid for 2 h, and destained by 10% (v/v) acetic acid. The band intensities on gel were analyzed by Bio‐Rad Image Lab Software.

### Statistical analysis

Non‐parametrical Kruskal–Wallis analysis was performed. The least significant difference test was employed to determine differences between means at a 5% significance level. Results were analyzed using the Statistical Software Package for Windows PASW Statistic 20.0 (SPSS, Chicago, IL).

## Results and Discussion

### Sensory analysis

Standards used in the sensory evaluation are listed in Table 1. Results listed in Table 2 showed no significant difference in scores of samples stored for less than 8 h, while significant differences (*P* < 0.05) in scores of samples stored for more than 8 h. There were significant differences between scores of samples stored for 12 h and 24 h, which meant sensory properties changed a lot from 12 h to 24 h. Scores of samples stored for 8 hours were about 10 points than that of samples stored for 12 h when taking each one of the six sensory properties into consideration. This showed sensory quality of soya milk drop a lot when its storage time was more than 8 h under room temperature. Thus, it is suggested that soya milk stored under room temperature should be sold out within 8 h.

**Table 1:** Sensory evaluation standards

	1–20 score	21–40 score	41–60 score	61–80 score	81–100 score
Color	Very poor:Uneven color, there are differences with soya milk color obviously	Poor:Uneven color, different from normal soya milk, bluish white color, or similar with water, uneven after shaking	General:Color slightly uneven or flat after shaking, and soya milk is close to normal	Good:Color uniformity, with pale yellow	Very good:According to panelists point of view, that color of soya milk is good
Bean odor	Very poor:No fragrance, there may be other smells	Poor:Can smell the aroma is extremely weak or no aroma, or have other scents coexist	General:Can smell the aroma, very weak or no scent, no peculiar smell	Good:Fragrance, suitable for drinking, no peculiar smell	Very good:strong aroma beans, after opening the bottle of bean aroma
Beany	Very poor:Have obvious gamey smell, may be related to the corruption of other smell coexist.	Poor:Have gamey smell, may be associated with bad breath, or fragrance	General:Gamey smell is not obvious, light scent, acceptable	Good:No gamey smell or very weak	Very good:No gamey smell
Quality	Very poor:Soybean Milk precipitation or bean dregs, Tofu pudding‐like,flocks, hand pinch significantly different size particles.	Poor:Soya milk appear little or no precipitation, hand knead particles are not obvious	General:No precipitation, hand knead slightly grainy	Good:No precipitation, hand knead slightly grainy or no particles	Very good:No precipitation, hand knead slightly grainy or no particles
Flavor	Very poor:Sour taste is obvious,bean dregs fermentation corruption flavorappeared	Poor:No acid odor, no pollution and corruption, but the flavor is not acceptable	General:No acid odor, no pollution and corruption, but the flavor is acceptable	Good:No sour smell, with Soybean Milk typical flavor, flavor or after heating	Very good:No acid odor, flavor, or heated flavor
Overall acceptability	Very poor:After opening the bottle, the panelists cannot accept it	Poor:The panelists can be detected, that can't be drinking	General:The panelists can drink, it is not recommended to sell	Good:The panelists can drink, can be sold	Very good:All the indicators are outstanding,the panelists can drink, it can be sold

**Table 2:** Sensory analysis

Sensory properties	Time (h)				
	0	4	8	12	24
Color	96.22 ± 1.56^a^	95.78 ± 1.48^a^	95.55 ± 1.33^a^	79.00 ± 2.50^b^	71.56 ± 2.70^c^
Bean odor	96.78 ± 1.56^a^	96.67 ± 1.50^a^	95.56 ± 2.12^a^	81.33 ± 1.66^b^	66.67 ± 2.30^c^
Beany	95.00 ± 2.18^a^	94.11 ± 2.47^a^	93.67 ± 2.00^a^	81.00 ± 1.87^b^	73.67 ± 2.24^c^
Quality	96.11 ± 1.45^a^	95.89 ± 2.08^a^	95.33 ± 1.12^a^	75.11 ± 2.37^b^	73.11 ± 1.97^b^
Flavor	94.22 ± 1.20^a^	93.89 ± 1.17^a^	93.44 ± 1.13^a^	72.11 ± 2.03^b^	64.00 ± 2.55^c^
Overall acceptability	93.56 ± 1.23^a^	93.22 ± 1.39^a^	92.89 ± 1.05^a^	71.78 ± 2.22^c^	71.22 ± 2.17^b^

Values are means ±standard error. For the sensory attributes, a 100‐point Hedonic scale was used (100 = like extremely, 1 = dislike extremely); the experiment was done in triplicate. Each time, 20 panelists involved. Values with different superscript letters within the same row differ significantly (*P* < 0.05).

### Effect of storage time on viscosity, pH, and bacteria of soya milk samples

**Figure 1:**
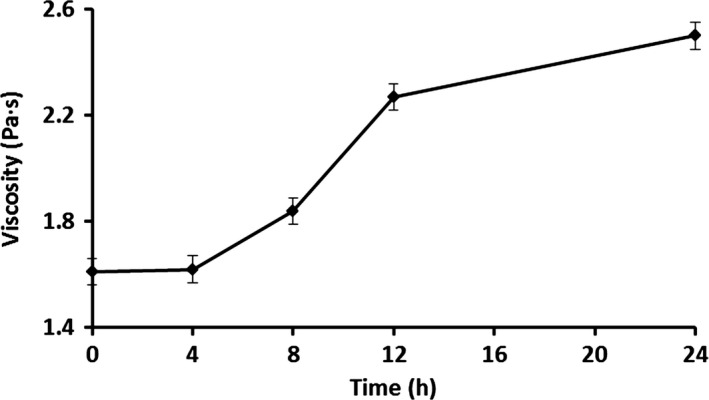
The viscosity changes of soya milk samples. Values expressed as means ±SE.

**Figure 2:**
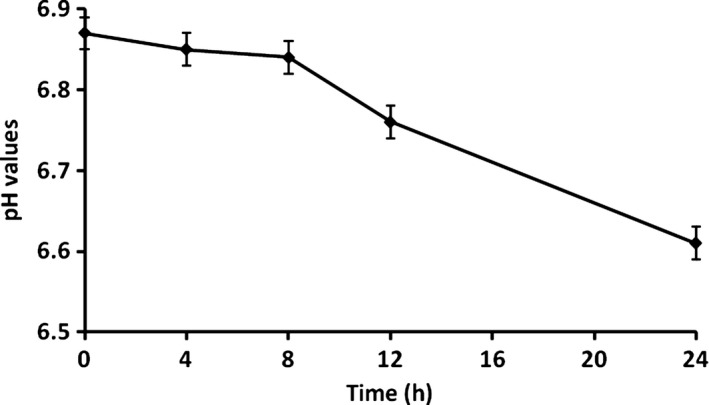
The pH value changes of soya milk samples. Values expressed as means ±SE.

**Figure 3:**
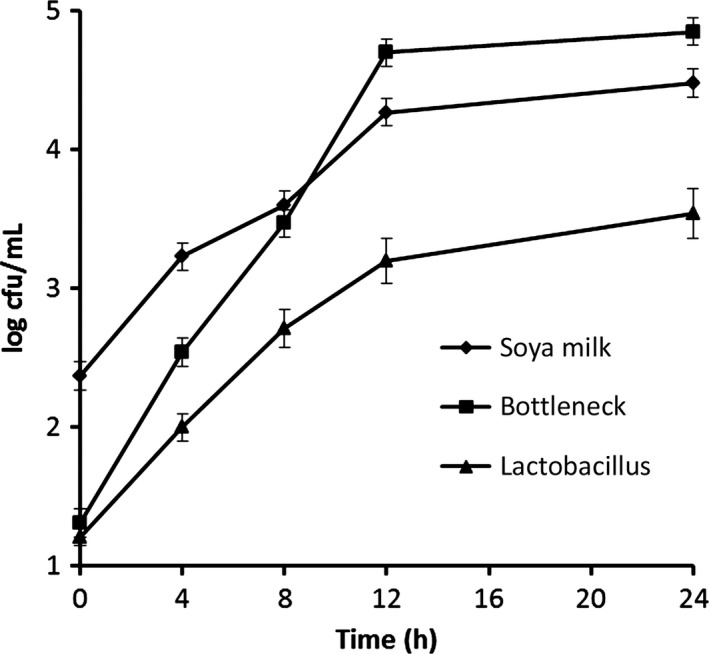
The bacteria analysis of soya milk samples. Values expressed as log cfu/mL ±SE.

The viscosity of a food system is dependent on the volume fraction occupied by the contributing particles in combination with the inherent viscosity of the continuous phase (Anema et al. 2014). The viscosity of soya milk samples increased continually during the storage time (Fig. 1). It was in a stable state within the first 8 h, and after 8 h, it increased quickly. As pH value is an important factor that may cause the volume fraction of casein micelles (Anema and Creamer 1993), we investigated the pH values during the experimental time. The pH value decreased from 6.87 to 6.61 during the storage time, and there was an obvious drop after 8 h (Fig. 2). Thus, the soya quality of milk was basically stable in 8 h. For the concentrate from the treated soya milk, the pH decreased the consistency coefficients of the soya milk concentrate during the storage time. This is also consistent with previous studies on the apparent viscosity of soya milk concentrates (Snoeren et al. 1984; van Hooydonk et al. 1986). Thus, changes of bacteria in soya milk and bottleneck were investigated. Figure 3 shows obviously that the total bacterial count both in soya milk and bottleneck, and *Lactobacillus* count in soya milk increased with storage time. The increasing rates were fast within 8 h and became moderate after 12 h. Total bacterial count in bottleneck reached 4.7 log CFU/mL, which is the limit detection for milk according to Guangdong enter‐exit inspection and quarantine compiled (2002) (Fig. 3). *Lactobacillus* was dominant bacteria in soya milk, which were more than 50% of the total bacteria count (Fig. 3). That means, the storage time should be less than 8 hours when soya milk is stored under 25 ± 2°C.

### Phylogenetic analysis

**Figure 4:**
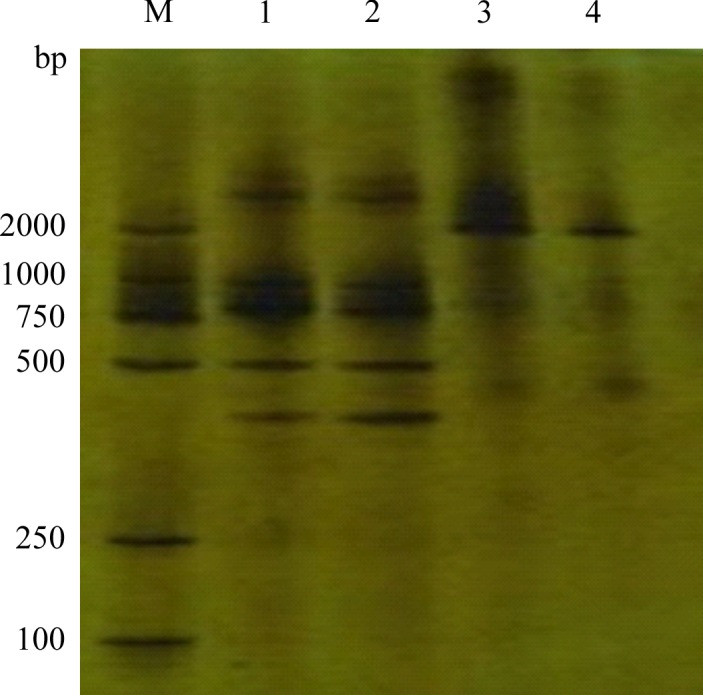
DGGE profiles of bacteria agarose gel electrophoresis bands recovered samples in the soya milk. (M) maker, (1) V3 area primers gram‐positive bacteria DNA bands, (2) V3 area primers gram‐negative DNA bands, (3) V3‐V5 area primers gram‐positive bacteria DNA bands, (4) V3‐V5 area primers gram‐negative DNA bands.

**Figure 5:**
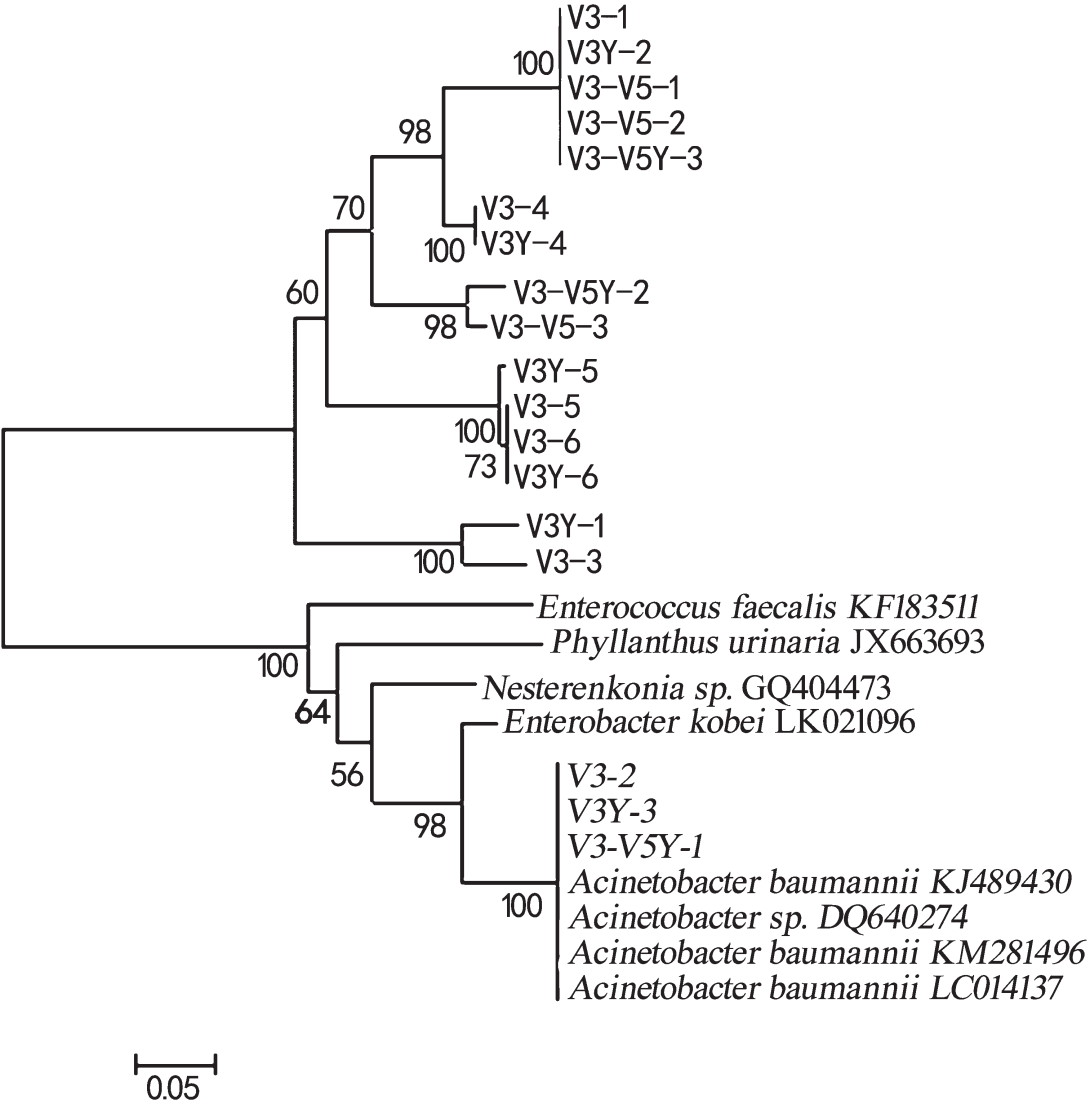
Phylogenetic tree analysis of the bacteria. The neighbor‐joining tree was constructed with a representative sequence of each OTU selected by the MOTHUR program. Numbers at the nodes indicate bootstrap values (expressed as a percentage of 1000 replications). Values in brackets represent the number of sequences found for each OTU. Symbols represent the distribution of the OTUs in the samples.

According to the results of the BLASTN algorithm in the GenBank database, eight strains representing homology higher 16S rDNA sequence bacterial were selected to compare with 18 tested strains. DGGE analysis was carried out to identify the *Lactobacillus* strains. Table 3 shows that closest relatives associated with hands in DGGE profile (Fig. 4), the results all verified that the credibility reached 99–100%. Under normal circumstances, the similarity of 16S rDNA sequences above 98% can be recognized as the same (Devereux et al. 1990). From the comparison results of the corresponding sequence of 18 strains bacteria 16S rDNA partial sequences in the GenBank, these bacteria can be divided into eight genera and 11 species. V3Y‐1 belongs to *Rummeliibacillus* genera, the bacteria is gram‐positive, facultative aerobic, and available in multiple environments. However, the current research on the bacteria of the genus is mainly theoretical research (Vaishampayan et al. 2009); V3Y‐2, V3Y‐3, V3‐1,V3‐2, V3‐ V5Y‐1, V3‐ V5Y‐3, V3‐ V5‐1, V3‐ V5‐2 belongs to *Acinetobacter* genera, *Acinetobacter baumannii* is a gram‐negative coccobacillus, known as the fifth most common etiological agent of nosocomial infections associated with intensive care unit residence (Senchenkova et al. 2015), and it is an important opportunistic pathogen and is often involved in various nosocomial infections, such as bacteremia, urinary tract infection, secondary meningitis, surgical site infection, and nosocomial and ventilator‐associated pneumonia, especially in patients who are admitted to intensive care and burn units (Peleg et al. 2008). For instance, 69.9% mortality has been demonstrated recently as a result of bacteremia caused by imipenem‐resistant A. *baumannii*. V3Y‐4, V3‐3, V3‐4 belongs to *Enterobacter* genera, *Enterobacter* as microbial contaminant in yoghurts manufactured from cow's milk and soymilk (Canganella et al. 1999); V3Y‐5, V3Y‐6 belongs to *Phaseolus* genera; V3‐5 belongs to *Phyllanthus* genera; V3‐6 belongs to *Bergia* genera; V3‐ V5Y‐2 belongs to *Zhihengliuella* genera; V3‐V5‐3 belongs to *Nesterenkonia* genera, *Nesterenkonia* genera is the bacteria produce amylase (Shafiei et al. 2010). Using MEGA 5.0 software to construct the bacterial phylogenetic tree (Fig.5), the bootstrap is 1000. The phylogenetic tree shows that V3‐1, V3Y‐2, V3‐V5‐1, V3‐V5‐2,V3‐V5Y‐3 formed a branch, verify the credibility reached 100%; V3‐4, V3Y‐4 formed a branch, verify the credibility reached 100%; V3‐V5Y‐2, V3‐V5‐3 formed a branch, verify the credibility reached 98%; V3Y‐5, V3‐5, V3‐6, V3Y‐6 formed a branch, verify the credibility reached 100%; V3Y‐1, V3‐3 formed a branch, verify the credibility reached 100%; V3‐2, V3Y‐3, V3‐V5Y‐1 and *Acinetobacter baumannii* formed a branch, verify the credibility reached to 100%.

**Table 3:** Closest relatives associated with hands in DGGE profile

Bands no.	Comparison results	GenBank sequential extraction	Similarity (%)
V3Y‐1	*Rummeliibacillus pycnus*	KM378586	99
V3Y‐2	*Acinetobacter baumannii*	KM281496	100
V3Y‐3	*Acinetobacter baumannii*	LC014137	100
V3Y‐4	*Enterobacter kobei*	LK021096	100
V3Y‐5	*Phaseolus vulgaris*	AJ007448	99
V3Y‐6	*Phaseolus vulgaris*	AJ007448	100
V3‐1	*Acinetobacter baumannii*	JQ066781	100
V3‐2	*Acinetobacter oleivorans*	KM391946	100
V3‐3	*Enterococcus faecalis*	KF183511	100
V3‐4	*Enterobacter ludwigii*	KM349408	100
V3‐5	*Phyllanthus urinaria*	JX663693	99
V3‐6	*Bergia texana*	JX663661	99
V3‐V5Y‐1	*Acinetobacter baumannii*	KJ489430	99
V3‐V5Y‐2	*Zhihengliuella salsuginis*	AB778264	99
V3‐V5Y‐3	*Acinetobacter sp*.	JQ912622	99
V3‐V5‐1	*Acinetobacter baumannii*	FR774572	99
V3‐V5‐2	*Acinetobacter sp*.	DQ640274	100
V3‐V5‐3	*Nesterenkonia sp*.	GQ404473	99

### SDS‐PAGE analysis of protein samples

**Figure 6:**
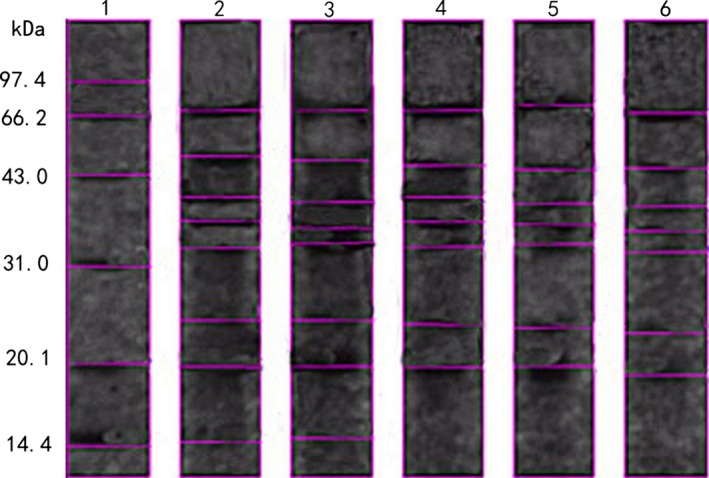
The SDS–page profiles of protein samples. (1) Marker; (2) Stored for 0 h sample; (3) Stored for 4 h sample; (4) Stored for 8 h sample; (5) Stored for 12 h sample; (6) Stored for 24 h sample.

The SDS–PAGE profiles of protein samples stored for 0 h, 4 h, 8 h, 12 h, and 24 h are shown in Figure 6. BIO‐BEST 200 E gel imaging system was used to analyze the protein in the treated soya milk samples (Fig. 6). The corresponding subunit molecular weight was shown in details in Table 4. Migration rate and the molecular weight of the protein showed a negative correlation between logarithmic in SDS‐PAGE gel electrophoresis system. Proteins of greater molecular weight showed smaller mobility. Comparison of these five sample bands, there was a subunit composition gap between samples. Soya milk protein contained 7–9 subunits and subunit molecular weight in the range of 13.08‐–106.8 kDa, while <3% in total protein content distributed among 90–107 kDa. Most of subunit molecular weight distributed from 20 to 90 kDa (Table 4). This shows that the treated soya milk protein samples mainly contain medium and small molecular weight subunit. It also shows the subunit in soya milk protein changed constantly during storage. And along with the extension of storage time, the amount of *Lactobacillus* in soya milk increased, the pH values decreased, leading to protein denaturation, and produce high‐molecular weight subunit (Jarpa‐Parra et al. 2014).

**Table 4:** Comparison with each subunit's molecular weight in different treated soya milk samples

Subunit No.	Molecular weight (kDa)	0 h	4 h	8 h	12 h	24 h
1	>97.4					
2	66.2~97.4	70.247	70.247	70.247	74.671	68.172
3	43.0~66.2	49.054	47.628	45.830	44.564	44.978
4	31.0~43.0	40.049	39.356	40.049	39.183	38.837
		36.940	35.910	36.768	36.424	35.567
		33.354	33.693	33.523	33.862	32.847
5	20.1~31.0	24.479	24.479	24.040	23.608	23.044
6	14.4~20.1	19.985	19.871	19.759	19.871	19.107
		14.634	14.868			

## Conclusions

Collectively, we used quantitative descriptive analysis to describe the change of sensory quality, viscosity, pH, protein composition, and bacteria in both soya milk and bottleneck, in commercial soya milk stored for 0, 4, 8, 12, and 24 h at 25 ± 2°C. The results show that with the extension of storage time, the above indicators are changed in the direction of the soya milk deterioration. The contamination may be the main reason for soya milk's deterioration. The recommendation is that the best shelf life of commercial soya milk is less than 8 h at room temperature. These results may help both factories and consumers to better understand the quality control of soya milk products.

## Conflict of Interest

None declared.

## References

[fsn3371-bib-0001] Anema, S. G. , and L. K. Creamer . 1993 Effect of the A and B variants of bothalpha S1‐ and kappa‐casein on bovine casein micelle solvation and kappa‐casein content. J. Dairy Res. 60:505–516. 8294607 10.1017/s0022029900027862

[fsn3371-bib-0002] Anema, S. G. , E. K. Lowe , S. K. Lee , and H. Klostermeyerl . 2014 Effect of the pH of skim milk at heating on milk concentrate viscosity. Int. Dairy J. 39:336–343.

[fsn3371-bib-0003] Bae, H. , S. H. Kim , M. S. Kim , R. C. Sicher , D. Lary , M. D. Strem , et al. 2008 The drought response of Theobroma cacao (cacao) and the regulation of genes involved in polyamine biosynthesis by drought and other stresses. Plant Physiol. Biochem..: PPB / Societe francaise de physiologie vegetale. 46: 174–188. 18042394 10.1016/j.plaphy.2007.10.014

[fsn3371-bib-0004] Burkus, Z. 2003 Determination of the molecular weight of barley *β*‐glucan using intrinsic viscosity measurements. Carbohydr. Polym. 54:51–57.

[fsn3371-bib-0005] Canganella, F. , M. L. Nespica , D. Giontella , and L. D. Trovatelli . 1999 Survival of *Enterobacter cloacae* and Pseudomonas paucimobilis in yoghurts manufactured from cow's milk and soymilk during storage at different temperatures. Microbiol. Res. 154:15–21. 10356792 10.1016/S0944-5013(99)80029-9

[fsn3371-bib-0006] Devereux, R. , S. H. He , C. L. Doyle , S. Orkland , D. A. Stahl , J. Legall , et al. 1990 Diversity and origin of Desulfovibrio species: phylogenetic definition of a family. J. Bacteriol. 172:3609–3619. 2361938 10.1128/jb.172.7.3609-3619.1990 PMC213334

[fsn3371-bib-0007] Fujimoto, C. , H. Maeda , S. Kokeguchi , S. Takashiba , F. Nishimura , H. Arai , et al. 2003 Application of denaturing gradient gel electrophoresis (DGGE) to the analysis of microbial communities of subgingival plaque. J. Periodont Res. 38:440–445. 12828664 10.1034/j.1600-0765.2003.02607.x

[fsn3371-bib-0008] Ghosh, D. , and P. Chattopadhyay . 2012 Application of principal component analysis (PCA) as a sensory assessment tool for fermented food products. J. Food Sci. Technol. 49:328–334. 23729852 10.1007/s13197-011-0280-9 PMC3614048

[fsn3371-bib-0009] Guangdong enter‐exit inspection and quarantine compiled . 2002 The domestic and foreign technical regulations and standards of food microorganism limit. China Standard Press, Beijing.

[fsn3371-bib-0010] van Hooydonk, A. C. M. , H. G. Hagedoorn , and I. J. Boerrigter . 1986 pH‐induced physico‐chemical changes of casein micelles in milk and their effect on renneting. 1. Effects of acidification on physico‐chemical properties. Neth. Milk Dairy J. 40:281–296.

[fsn3371-bib-0011] Jarpa‐Parra, M. , F. Bamdad , Y. Wang , Z. Tian , F. Temelli , J. Han , et al. 2014 Optimization of lentil protein extraction and the influence of process pH on protein structure and functionality. LWT ‐ Food Sci. Technol. 57:461–469.

[fsn3371-bib-0012] Jooyandeh, H. 2011 Soy products as healthy and functional foods. Middle‐East J. Sci. Res. 7:71–80.

[fsn3371-bib-0014] Kwok, K. C. , and K. Niranjan . 1995 Review: effect of thermal processing on soymilk. Int. J. Food Sci. Technol. 30:263–295.

[fsn3371-bib-0015] Li, Q. , Y. Hu , F. Chen , J. Wang , Z. Liu , and Z. Zhan . 2014 Environmental controls on cultivated soybean phenotypic traits across China. Agric. Ecosyst. Enviro. 192:12–18.

[fsn3371-bib-0016] Marangoni, F. , and A. Poli . 2010 Phytosterols and cardiovascular health. Pharmacol. Res. 61:193–199. 20067836 10.1016/j.phrs.2010.01.001

[fsn3371-bib-0017] Niki, E. , and N. Noguchi . 2004 Dynamics of antioxidant action of Vitamin E. Acc. Chem. Res. 37:45–51. 14730993 10.1021/ar030069m

[fsn3371-bib-0018] Odu, N. N. , and N. N. Egbo . 2012 Assessment of the effect of different preservatives on the keeping quality of soymilk stored at different temperatures. J. Nat. Sci. 10:1–9.

[fsn3371-bib-0019] Patisaul, H. B. , and W. Jefferson . 2010 The pros and cons of phytoestrogens. Front. Neuroendocrinol. 31:400–419. 20347861 10.1016/j.yfrne.2010.03.003 PMC3074428

[fsn3371-bib-0020] Peleg, A. Y. , H. Seifert , and D. L. Paterson . 2008 *Acinetobacter baumannii*: emergence of a successful pathogen. Clin. Microbiol. Rev. 21:538–582. 18625687 10.1128/CMR.00058-07 PMC2493088

[fsn3371-bib-0021] Purwandari, U. , N. P. Shah , and T. Vasiljevic . 2007 Effects of exopolysaccharide‐producing strains of Streptococcus thermophilus on technological and rheological properties of set‐type yoghurt. Int. Dairy J. 17:1344–1352.

[fsn3371-bib-0022] Reilly, J. K. , A. J. Lanou , N. D. Barnard , K. Seidl , and A. A. Green . 2006 Acceptability of soymilk as a calcium‐rich beverage in elementary school children. J. Am. Diet. Assoc. 106:590–593. 16567156 10.1016/j.jada.2006.01.010

[fsn3371-bib-0023] Schägger, H. 2006 Tricine–SDS–PAGE. Nat. Protoc. 1:16–22. 17406207 10.1038/nprot.2006.4

[fsn3371-bib-0024] Senchenkova, S. N. , A. S. Shashkov , A. V. Popova , M. M. Shneider , N. P. Arbatsky , K. A. Miroshnikov , et al. 2015 Structure elucidation of the capsular polysaccharide of *Acinetobacter baumannii* AB5075 having the KL25 capsule biosynthesis locus. Carbohydr. Res. 408:8–11. 25816997 10.1016/j.carres.2015.02.011

[fsn3371-bib-0025] Shafiei, M. , A. A. Ziaee , and M. A. Amoozegar . 2010 Purification and biochemical characterization of a novel SDS and surfactant stable, raw starch digesting, and halophilic *α*‐amylase from a moderately halophilic bacterium, *Nesterenkonia* sp. strain F. Process Biochem. 45:694–699.

[fsn3371-bib-0026] Snoeren, T. H. M. , J. A. Brinkhuis , A. J. Damman , and H. J. Klok . 1984 Viscosity and age‐thickening of skim‐milk concentrate. Neth. Milk Dairy J. 38:43–53.

[fsn3371-bib-0027] Tamura, K. , D. Peterson , N. Peterson , G. Stecher , M. Nei , and S. Kumar . 2011 Molecular evolutionary genetics analysis using maximum likelihood, evolutionary distance, and maximum parsimony methods. Mol. Biol. Evol. 28:2731–2739. 21546353 10.1093/molbev/msr121 PMC3203626

[fsn3371-bib-0028] Vaishampayan, P. , M. Miyashita , A. Ohnishi , M. Satomi , A. Rooney , M. T. Laduc , et al. , et al. 2009 Description of *Rummeliibacillus stabekisii* gen. nov., sp. nov. and reclassification of Bacillus pycnus Nakamura, 2002 as *Rummeliibacillus pycnuscomb*. nov. Int. J. Syst. Evol. Microbiol. 59:1094–1099. 19406799 10.1099/ijs.0.006098-0

[fsn3371-bib-0029] Yu, Z. M. , and M. Orrison . 2004 Comparisons of different hypervariable regions of rrs genes for use in fingerprinting of microbial communities by PCR denaturing gradient gelelectrophoresis. Appl. Environ. Microbiol. 70:4800–4806. 15294817 10.1128/AEM.70.8.4800-4806.2004 PMC492348

[fsn3371-bib-0030] Zijnge, V. , J. M. Harmsen , J. W. Kleinfelder , M. E. Vanderrest , J. E. Degener , and G. W. Welling . 2003 Denaturing gradient gel electrophoresis analys is to study bacterial community structure in pockets of periodontitis patients. Oral Microbiol. Immunol. 18:59–65. 12588461 10.1034/j.1399-302x.2003.180110.x

